# Fast and efficient short read mapping based on a succinct hash index

**DOI:** 10.1186/s12859-018-2094-5

**Published:** 2018-03-09

**Authors:** Haowen Zhang, Yuandong Chan, Kaichao Fan, Bertil Schmidt, Weiguo Liu

**Affiliations:** 1 School of Software, Shandong University, Shunhua Road 1500, Jinan, Shandong China; 2 Laboratory for Regional Oceanography and Numerical Modeling, Qingdao National Laboratory for Marine Science and Technology, Qingdao, 266237 Shandong China; 3 School of Computational Science and Engineering, Georgia Institute of Technology, Atlanta, 30332 GA USA; 4 Johannes Gutenberg University, Mainz, Germany

**Keywords:** Next-generation sequencing, Read mapping, Hash index, Seed selection

## Abstract

**Background:**

Various indexing techniques have been applied by next generation sequencing read mapping tools. The choice of a particular data structure is a trade-off between memory consumption, mapping throughput, and construction time.

**Results:**

We present the succinct hash index – a novel data structure for read mapping which is a variant of the classical *q*-gram index with a particularly small memory footprint occupying between 3.5 and 5.3 GB for a human reference genome for typical parameter settings. The succinct hash index features two novel seed selection algorithms (group seeding and variable-length seeding) and an efficient parallel construction algorithm, which we have implemented to design the FEM (Fast(F) and Efficient(E) read Mapper(M)) mapper. FEM can return all read mappings within a given edit distance. Our experimental results show that FEM is scalable and outperforms other state-of-the-art all-mappers in terms of both speed and memory footprint. Compared to Masai, FEM is an order-of-magnitude faster using a single thread and two orders-of-magnitude faster when using multiple threads. Furthermore, we observe an up to 2.8-fold speedup compared to BitMapper and an order-of-magnitude speedup compared to BitMapper2 and Hobbes3.

**Conclusions:**

The presented succinct index is the first feasible implementation of the *q*-gram index functionality that occupies around 3.5 GB of memory for a whole human reference genome. FEM is freely available at https://github.com/haowenz/FEM.

## Background

DNA sequencing has become a powerful technique in many areas of biology and medicine. Technological breakthroughs in high-throughput sequencing platforms during the last decade have triggered a revolution in the field of genomics. Up to billions of short reads can be quickly and cheaply generated by these platforms in a single run, which in turn increases the computational burden of genomic data analysis. The first step of most associated pipelines is the mapping of the generated reads to a reference genome.

Read mappers fall into one of the two classes. One class, including FastHASH [1], mrsFAST [2], RazerS3 [3], BitMapper [4], and Hobbes [5], is referred to as *all-mappers*. All-mappers attempt to find all mapping locations of each read. The other class, including Bowtie2 [6], BWA [7], and GEM [8], is referred to as *best-mappers*. Best-mappers use some heuristic methods for identifying one or a few top mapping locations for each read. These heuristic strategies can lead to a significant improvement in speed. However, for some specific applications, such as CHIP-seq experiments [9], copy number variation and RNA-seq transcript abundance quantification [10], it is often more desirable to use all-mappers to identify all mapped locations of each read. In this work, we focus on designing an efficient and scalable all-mapper algorithm.

To simplify searching the whole reference which contains billions of characters, all-mappers often use the seed-and-extend strategy. Using this strategy, all-mappers initially index fixed-length seeds or *k*-mers (substrings of length *k*) of the reference genome into a hash table or similar data structure. Secondly, based on the observation that every correct mapping for a read in the reference genome will also be mapped by the seed, each query read is divided into seeds to query the hash table index for candidate mapping locations. Finally, dynamic programming algorithms such as Needleman-Wunsch [11] and Smith-Waterman [12] are used to extend the read at each candidate location and verify the correctness of each candidate location below a given error threshold *e*.

A number of indexing techniques have been applied for the read mapping problem. These include suffix trees [13], suffix arrays [14], Burrows-Wheeler transform (BWT) with FM-index [15], and *q*-grams [16–18]. The choice of the index is key to performance. State-of-the-art all-mappers mainly rely on the *q*-gram index which typically occupies around 12GB of memory for a human reference genome. Since this index typically has to be kept in main memory during the mapping process, approaches with a much smaller memory footprint are highly desirable. This is particular important for modern computer architectures featuring fast memory of limited size such as high bandwidth memory (HBM).

### Short read alignment

*Short-read alignment* (SRA) is a crucial component of almost every NGS pipeline. The goal of SRA is to map each read to the true location in the given reference genome. Note that this location might neither be unique (because of repeat structures in the reference genome) nor be an exact match (because of sequencing errors or true genomic variations). From a computational perspective, we can formulate SRA as an approximate sequence matching problem as follows.

#### **Definition 1**

*(Edit distance)* The *edit (or Levenshtein) distance* between two sequences *S*_1_ and *S*_2_ over the alphabet *Σ* is the minimum number of point mutations (i.e. insertions, deletions, or substitutions) required to transform *S*_1_ into *S*_2_.

#### **Definition 2**

*(Short-read alignment)* Consider a set of reads ${\mathcal {R}}$, a reference genome *G*, and an error threshold *e*. Find all substrings *g* of *G* that are within edit distance *e* to some read $R \in {\mathcal {R}}$. We call such occurrences *g* in *Gmatches*.

SRA can be solved by a classical dynamic programming (DP) approach which calculates the semi-global alignment between each $R \in {\mathcal {R}}$ and *G*. Unfortunately, the resulting time complexity proportional to the product of sequence lengths per alignment renders the alignment of a large number of short reads to a mammalian reference genome intractable.

To address this problem most state-of-the-art solutions are based on a *seed-and-extend* approach consisting of two phases: the first phase identifies promising candidate regions (seeds) for each read in *G* while the second phase determines whether a seed can actually be extended to a full match [19]. Implementations of the first phase are usually based on the algorithmic ideas of *indexing* and *filtering*. A possible filtering strategy in order to discard large regions of *G* is based on the pigeonhole principle. Applied to the SRA scenario, the pigeonhole lemma states that if a read $R \in {\mathcal {R}}$ is divided into *e*+1 non-overlapping *q*-grams (substrings of length *q*=⌊|*R*| /(*e*+1)⌋), then at least one of them occurs exactly in a match. Such exact occurrences can be identified quickly by storing *G* in an appropriate *q*-gram index data structure. In practice, some SRA tools also use more advanced methods to find seeds such as *q*-gram counting. The subsequent extension stage requires the implementation of a *verification* algorithm in order to determine whether an actual match (with an edit distance ≤*e*) actually exists in the vicinity of each seed location. Current SRA tools apply fast and parallelized versions of DP based algorithms for this step such as the Smith-Waterman algorithm.

### Hash index data structure

For a sequence *s*, we denote the substring that begins at position *a* and ends at position *b* as *s*[*a*..*b*]. We use |*s*| to denote the length of *s*. For any *k*-mer *s*_1_, we denote its occurrence list and the length of the list as *L*(*s*_1_) and |*L*(*s*_1_)|, respectively.

**Fig. 1:**
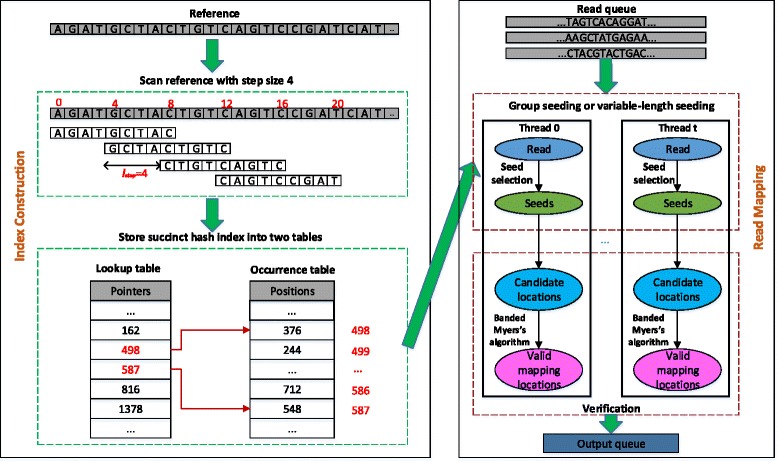
Workflow of FEM

The traditional hash index stores all occurrences for each *k*-mer (i.e. the locations the *k*-mer occurs in the reference genome). As shown in Fig. 1, this hash index consists of two (dense) tables, lookup table *Lu* and occurrence table *Occ*. Each element in *Lu* stores the start index of the occurrence list of its corresponding *k*-mer in the reference genome in *Occ*. *Occ* stores the list of locations for every *k*-mer in ascending order.

The number of entries in *Lu* is 4^*k*^. Thus, its size grows exponentially with *k*. However, the frequencies of *k*-mers decrease when employing larger *k* [20]. Typically, the values of *k* utilized by SRA tools usually range between 10 to 13. Thus, *Lu* exhibits a relatively low memory footprint, ranging from 1MB to 64MB.

Since *Occ* needs to record the occurrence lists of all *k*-mers in a given reference genome sequence *G*, it needs to store |*G*|−*k*+1 positions. Assuming that each position can be represented by an integer, the *size* of a traditional hash index is the sum of the size of *Lu* and *Occ*, which equals to

1 $$ {size} = SOI \times (4^{k} + \left |G \right | - k +1).  $$

*SOI* denotes the size of integer in bytes. For larger reference genomes |*G*| dominates 4^*k*^. In this case the *size* of a hash index approximately equals to the size of *Occ*, which is

2 $$ {size} \approx SOI \times (\left |G \right | - k +1).  $$

### Related work

There have been a variety of techniques proposed for solving the SRA problem. The majority of all-mappers is based on a filtration plus validation approach. Many state-of-the-art seed selection algorithms aim at reducing the sum of seed frequencies of a read using different heuristics or greedy algorithms in the filtration stage. Existing seed selection algorithms can be classified into three categories:

1. Extend frequent seeds in order to reduce their occurrences. The *adaptive seeds filter* used in the GEM read mapper [8] belongs to this category. LAST [21] also uses adaptive seeds for read mapping and genome comparison.
2. Sample the frequency of each seed and choose seeds with low frequencies. Both *cheaper k-mer selection* (CKS) used in FastHASH [1] and *optimal prefix selection* (OPS) used in Hobbes [5] belong to this category. For a fixed seed length *k* and a read of length *L*, CKS samples $\lfloor \frac {L}{k}\rfloor $ seed positions in a read, the interval between consecutive positions is *k* base-pairs. Different from CKS, the OPS algorithm allows for a greater freedom of choosing seed positions; i.e. each seed can be selected from any position in the read. Although OPS is more complex, it is capable of finding less frequent seeds compared to CKS.
3. Discover the least frequently-occurring set of seeds by a DP-based algorithm. The *optimal seed solver* (OSS) algorithm [20] belongs to this type. Currently, the OSS algorithm has not been integrated into existing read mappers due to significant overheads in terms of both memory and computation.

For the validation stage, a variety of DP-based alignment algorithms can be used to calculate the edit distance between a read and a reference candidate region. The Needleman Wunsch [11] algorithm for global alignment and the Smith-Waterman algorithm [12] for local alignment can be used in the validation stage. However, the speed of these is insufficient. Myers algorithm [22] is more efficient by exploiting bit-parallelism. It encodes a whole DP column in terms of two bit-vectors and computes the adjacent column using 17 bit-wise operations. RazerS3 [3] implements a banded version of Myers algorithm. The latest version of RazerS3 further accelerates the banded Myers algorithm by SIMD vectorization using SSE instructions. More recently, BitMapper [4] and BitMapper2 [23] have been proposed for improving candidate verification. They verify multiple candidate positions at the same time using a variation of Myers’ bit vector verification algorithm.

## Methods

In this section, we first present the succinct hash index together with a parallel construction algorithm. We illustrate how it can reduce the index size. Subsequently, we propose two new seed selection algorithms called group seeding and variable-length seeding based on the succinct hash index. We show how they guarantee to return all mappings under hamming distance and edit distance, respectively. Finally, we demonstrate the workflow of FEM, a novel read mapper which adopts these concepts.

### Succinct Hash Index

As mentioned in “Hash index data structure” section, the traditional hash index stores all locations of the occurrence for all possible *k*-kmers. For larger reference genomes, this requires a large amount of memory. For example, for a human reference genome consisting of more than 3G base-pairs (bps), it needs more than 12GB to load its hash index into memory according to Eq. 2. However, for even larger genomes such as the wheat reference genome containing about 16G bps, the traditional hash index requires more than 64GB memory. Furthermore, the construction of the traditional hash index requires a complete scan of the reference genome sequence leading to long construction times.

To reduce the memory consumption for read mapping and the run time for index construction, we present a new index data structure called succinct hash index. The key idea of the succinct hash index is inspired by the FM-index [7], which only keeps a small portion of entries of the suffix array and retrieves the discarded entries with the help of nearby known entries.

**Fig. 2:**
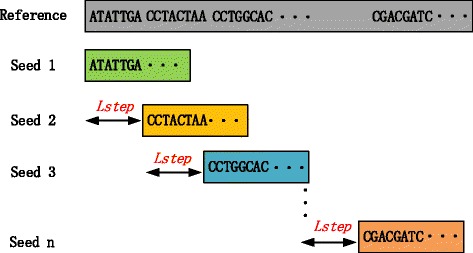
Example of seeding for index construction of the succinct hash index using *l*_*step*_=7

Different from the traditional hash index, the succinct hash index only stores the locations which are a multiple of *l*_*step*_ in the occurrence list *Occ*. Here, *l*_*step*_ is the step size for scanning the reference genome sequence. Figure 2 illustrates the construction progress using *l*_*step*_=7. When building the traditional hash index, to retrieve all occurrences for each *k*-mer during mapping, *l*_*step*_ is always set to one to record all locations. However, the succinct hash index employs *l*_*step*_ larger than one. Thus, the size of *Lu* does not change but the size of *Occ* is reduced by the factor of *l*_*step*_, i.e.

3 $$ SOI \times \frac{\left |G \right | - k +1}{l_{step}}.  $$

For a human reference genome, the size of its succinct hash index is only about 3GB for *l*_*step*_=4.

Since the succinct hash index does not scan and save all locations of the reference genome sequence, we miss locations which are not a multiple of *l*_*step*_ when trying to retrieve them. We call those locations *missed locations* and will show how to handle them with two new seed selection algorithms later.

In order to further accelerate index construction, we have designed a novel parallel index construction algorithm. Instead of directly inserting locations for each *k*-mer into its location list, we temporarily store a pair for each *k*-mer which contains its hash value and occurred location into an auxiliary table. This process can be parallelized using multiple threads. Subsequently, we sort this auxiliary table by the hash value of each pair and then by locations for pairs with the same hash value. We take advantage of the parallel sort primitive of the Intel TBB library to accelerate this process. Finally, we count the occurrence for every *k*-mer and build the two tables *Lu* and *Occ*. Algorithm 1 describes this parallel algorithm in detail. Since the first two steps are predominant in the whole process, the algorithm has good scalability with respect to the number of utilized threads.



### Group seeding

The key idea of traditional seed selection algorithms is based on the pigeonhole principle. Given an error threshold *e*, they select *e*+1 non-overlapping seeds. Due to the pigeonhole principle, at least one *k*-mer will not be affected by errors. Thus all the occurrences of these *k*-mers can be retrieved as candidate locations to be verified later, which guarantees to find all mapping locations with at most *e* errors for each read.

However, usage of the succinct hash index can cause missed locations when retrieving occurrence lists for *k*-mers. Thus, we present a modified seed selection algorithm called group seeding, which can retrieve all candidate locations for reads with respect to hamming distance using a succinct hash index. Our new seed selection algorithm is based on the following two definitions:

#### **Definition 3**

*(Position groups)* We define a partition of the set of positions *P* in the given reference genome sequence into *l*_*step*_ mutually disjoint sets *P*_*i*_, 0≤*i*<*l*_*step*_ called *position groups*. *P*_*i*_, 0≤*i*<*l*_*step*_, contains all reference genome positions *p* with *p* mod *l*_*step*_=*i*. Thus, $P = \bigcup ^{l_{step}-1}_{i=0} P_{i}$.

#### **Definition 4**

*(Seed groups)* We define a partition of the set of all substrings of length *k* (seeds) of a read *R* (denoted as *S*^*k*^) into *l*_*step*_ sets $S^{k}_{i}$, 0≤*i*<*l*_*step*_, called *seed groups*. $S^{k}_{i}$ contains all seeds that start at a location *j* in *R*, 0≤*j*≤|*R*|−*k*, with *j* mod *l*_*step*_=*i*. Thus, $S^{k} = \bigcup ^{l_{step}-1}_{i=0} S^{k}_{i}$.

Using these definitions, we can formulate the following observation if we consider no indels in the alignment between reads and reference genomes.

#### **Lemma 1**

Consider a read *R* which is mapped to the reference genome at position *p* with *p* mod *l*_*step*_=*i*; i.e. *p*∈*P*_*i*_. Then only seeds belonging to seed group *S*_*j*_ of *R* can be retrieved from the succinct hash index with

4 $$ (i+j) = l_{step}.  $$

**Fig. 3:**
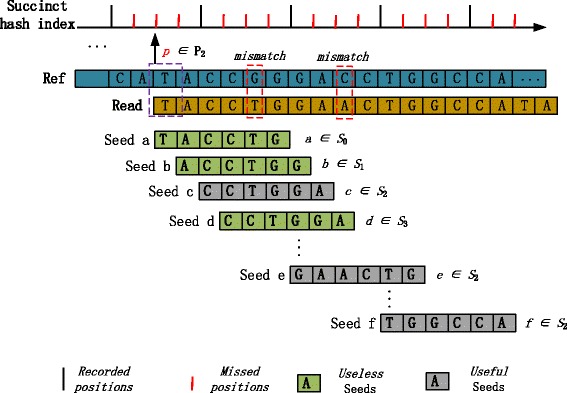
Consider a mapping position *p*∈*P*_2_ of a read *R* in a reference genome sequence. We distinguish useful and useless seeds in *R* for searching the mapping position *p* using *l*_*step*_=4

We illustrate the correctness of Lemma 1 using Fig. 3 as an example configuring the step-size *l*_*step*_ as 4 and considering a read *R* and a mapping location belonging to *P*_2_. In this case only seeds belonging to seed group *S*_2_ appear in a recorded location which can be retrieved from the succinct hash index. Since we assume that there are no insertions or deletions in the alignment, the seeds c, e, and f are in *S*_2_. Thus, all seeds in group *S*_2_ can be used to search for a position *p* in *P*_2_, whereby the sum of the position group index *i* and the seed group index *j* equals to the step-size *l*_*step*_.

Based on the definitions and Lemma 1, we design our group seeding algorithm based on a divide-and-conquer strategy tailored towards the succinct hash index as shown in Fig. 4. The basic idea of group seeding can be represented by three steps:

1. We divide all candidate mapping locations and all seeds in the read into *l*_*step*_ groups.

**Fig. 4:**
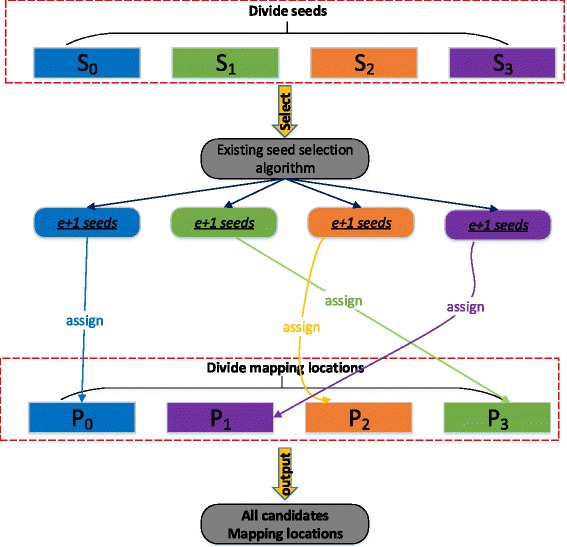
An illustration of retrieving all locations of a read with the group seeding algorithm using *l*_*step*_=4
2. Each position group *P*_*i*_,0≤*i*<*l*_*step*_, is assigned a specific seed group *S*_*j*_ according to Eq. 4.
3. Any existing seed selection algorithm can be used to select *e*+1 non-overlapping seeds from a specific seed group *S*_*j*_. These *e*+1 non-overlapping seeds are used to search the succinct hash index for all candidate mapping locations with respect to position group *P*_*i*_, where *i*+*j*=*l*_*step*_. The union of identified locations for each position group *P*_*i*_ forms the set of mapping candidates of a read *R*.

Group seeding supports any existing seed selection algorithm as long as it guarantees to find all candidate locations. In FEM, we utilize a combination of OPS [5] with an additional prefix algorithm [24] as the basic seed selection algorithm. The OPS algorithm is efficient since it aims to select a set of seeds with the minimal total number of candidate locations. Furthermore, the additional prefix algorithm can further decrease the number of candidate locations for any existing seed selection algorithm. The key idea is to retrieve all occurrences of *e*+2 seeds from the index and then select locations that come from at least two seeds as candidates. However, we need a modification of the original OPS algorithm since it uses a DP-based method to select *e*+1 non-overlapping seeds from a seed pool whereby seeds can start from any positions in the read. In order to integrate OPS into the group seeding algorithm, for a position group *P*_*i*_, we limit the seed pool of OPS to the associated seed group *S*_*j*_.

Since seed selection among different seed groups are independent from each other, group seeding can be efficiently parallelized on modern CPUs. Although group seeding guarantees to return all mapping locations when exclusively considering mismatches, it can maintain high accuracy if there are insertions or deletions. Group seeding guarantees no false negatives as long as the numbers of seeds in each seed groups after location *i* on read *R* are equal if an indel occurred at location *i*.

### Variable-length seeding

To tolerate indels, we propose *variable-length seeding* as another novel seed selection algorithm. Different from group seeding, variable-length seeding guarantees the return of all mapping locations when considering both mismatches and indels based on the succinct hash index. Let *k*^′^ equal to *k*+*l*_*step*_−1. The new seeding algorithm is based on the following definition:

#### **Definition 5**

*(Sub-seed)* Consider a seed *S* at least *k*^′^ base-pairs in length of a read *R*. We define any substring of length *k* of *S* as sub-seed $S^{s}_{i}$ if it occurs at location *i* in *S*.

Then variable-length seeding gets insight from Lemma 2.

#### **Lemma 2**

Given an error-free seed *S* of length *k*^′^, any of its occurrences on the reference genome can be retrieved by at least one of its sub-seeds.

**Fig. 5:**
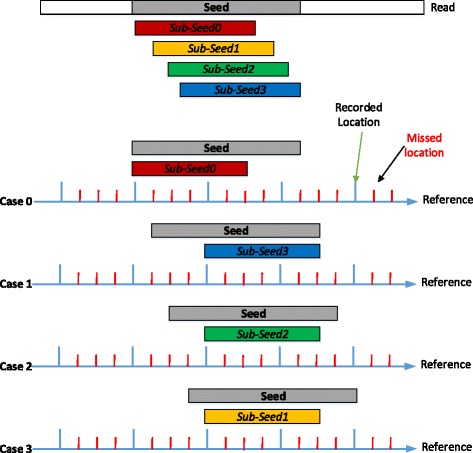
The length of the seed is 9 and the length of any sub-seeds of it is 6. Since *l*_*step*_=4, 4 sub-seeds of the seed are generated. The occurred position of the seed can belong to any of the 4 position groups, which is showed in four cases. In any case, the occurred location can be retrieved with one of its sub-seeds from the succinct hash index

In order to demonstrate the correctness of Lemma 2, we use the exhaustive method shown in Fig. 5. Given the seed *S* of length *k*^′^, we need to retrieve all locations where it occurs on the reference genome for the subsequent verification step. We first generate *l*_*step*_ sub-seeds from seed *S*. Without loss of generality, we use *p* to denote any position on the reference genome where *S* occurs. The succinct hash index records one every *l*_*step*_ positions. Thus, for positions *p*, *p*+1, … *p*+*l*_*step*_−1, one and only one of them is a recorded position denoted as *p*_*s*_=*p*+*i*, where 0≤*i*≤*l*_*step*_−1. Then, *p*_*s*_ is in the occurrence list of sub-seed $S^{s}_{i}$, i.e. $p_{s}\in L(S^{s}_{i})$, which can be retrieved.

Based on Lemma 2, we propose the basic idea of naive variable-length algorithm consisting of three steps:

1. We estimate the frequency of each seed of length *k*^′^ by accumulating the frequencies of its *l*_*step*_ sub-seeds.
2. Using an existing seed selection algorithm, we select a set of *e*+1 non-overlapping seeds with a minimal length of *k*^′^. We denote this set of seeds as *SSet*.
3. For each seed in *SSet*, we generate *l*_*step*_ sub-seeds to search the succinct hash index. The union of all locations retrieved by each sub-seed forms the set of all candidate mapping locations.

Naive variable-length seeding features an existing seed selection algorithm in the first step. Though each seed in *SSet* further generates *l*_*step*_ sub-seeds, the occurrences of the seeds do not increase significantly. Since the occurrences of the sub-seeds are also reduced due to the sub-sampling by means of the succinct hash index, the accumulated frequencies of the sub-seeds can be close to the frequency of the seed. However, by increasing the seed length to *k*^′^, this algorithm is limited to a smaller seed pool. Thus, the naive variable-length algorithm produces many candidate seeds which may decrease the efficiency of the subsequent verification stage.

A previous study [20] on seed frequency estimation shows how occurrences of a seed decrease when *k* grows larger. Inspired by this observation, we employ several strategies to extend the fixed-length seeds, i.e. seeds with length *k*^′^, to variable-length seeds in order to reduce candidate locations as follows.

1. We extend the seeds in *SSet* as long as they do not overlap with each other. Seeds with higher frequency compared to their neighboring seeds in *SSet* are extended with higher priority.
2. Within each extended seed *S*_*i*_, 0≤*i*≤*e*, all sub-seeds are divided into *l*_*step*_ groups called *sub-seed groups*. Two sub-seeds $S^{s_{i}}_{x}$ and $S^{s_{i}}_{y}$ are in the same sub-seed group if and only if their start locations *x* and *y* in *S*_*i*_ satisfy
  5 $$ x\bmod l_{step}=y\bmod l_{step}.  $$
3. For each sub-seed group, we choose the least frequent sub-seed. We use *BSet*_*i*_ to denote the set of chosen sub-seeds for *S*_*i*_. The set $BSet=\bigcup ^{e}_{i=0}BSet_{i}$ forms the set of all candidate mapping locations.

Algorithm 2 shows how the variable-length seeding algorithm generates candidate locations for read *R*.



In the first for-loop (Line 2), the frequency of each seed in *SSet* is estimated by the sum of frequencies of *l*_*step*_ sub-seeds. *Est*[*i*] stores the estimated frequency of a seed starting at location *i* of *R*. We utilize any existing seed selection algorithm to select a set of *e*+1 non-overlapping seeds and store them in *SSet* (Line 8). Each seed in *SSet* is represented by a four-tuple (*start,end,f,s*), where *start* and *end* denote the start and end location of the seed in *R*, respectively. *f* denotes the estimated frequency of the seed and *s* denotes the seed sequence.

We extend the first seed in *SSet* so that it starts from the first location in *R* (Line 9). Similar in Line 10 for the end of the last seed in *SSet* to the end of *R*. During the seed extension stage in the second for-loop (Line 11), seeds in *SSet* with higher frequency compared with their neighboring seeds in *SSet* are given higher extension priority. When the current seed is more frequent, we set its start to the end of the previous seed in *SSet*, which indicates that it is “extended" (Line 13). Otherwise, the previous seed in *SSet* occurs more frequently. In this case, we set the end of it to the start of current seed (Line 15).

We use a pair (*loc,f*) to represent each sub-seed, where *loc* is the location of the sub-seed in *R* and *f* is its frequency. After extending a seed *S*_*i*_ in *SSet*, for each sub-seed in *S*_*i*_, we find out which sub-seed group it belongs to (Line 25), its location on *R* (Line 26) and its frequency (Line 27). Then, the least frequent sub-seed is selected within each sub-seed group and stored in *BSet*_*i*_[*j*], where *j* denotes that the sub-seed belongs to sub-seed group *j*. A phasing on the length of the current seed is employed immediately after selecting the least frequent sub-seed by leaving its unused base pairs to the next seed in *SSet* (Line 35). Finally, we unite all the selected sub-seed sets (Line 38) and retrieve the occurred locations of all selected sub-seeds to formulate the candidate location list *CList* (Line 39).

Hobbes2 [24] proposed to select *e*+2 non-overlapping seeds for generating candidate positions and showed that adding an additional seed significantly reduces the number of candidates thus accelerating read mapping. Based on this observation, we also select *e*+2 seeds in our approach. According to Hobbes2, it is still reasonable to assume that each seed independently generates candidate positions when using *e*+2 seeds. Hence, we can select an optimal combination of *e*+2 instead of *e*+1 seeds in Algorithm 2 (Line 8).

Since the variable-length seeding algorithm has fully utilized the unused base-pairs between adjacent seeds, it allows a greater freedom of choosing sub-seed positions for each seed in *SSet* and thus generates less candidate locations compared to a naïve implementation.

### FEM workflow

The workflow of FEM is shown in Fig. 1. FEM is based on a seed-and-extend strategy and is targeted at standard multi-core CPUs and takes advantage of multi-threading as well as SIMD instructions to accelerate the mapping process. It employs a load balancing scheme implemented using the Pthreads library. After obtaining the reference genome sequence, FEM first constructs the succinct hash index to be used for the alignment. The left part of Fig. 1 presents the construction progress of the succinct hash index. After loading the index, reads are loaded into a read queue gradually. Multiple threads exclusively get reads from the read queue and map them back to the reference genome as shown in the right part of Fig. 1. The mapping process mainly consists of the following steps.

1. FEM retrieves candidate locations from the succinct hash index for each read with group seeding or variable-length seeding. In this step, we choose optimal prefix *q*-gram [5] as the seed selection algorithm and use additional *q*-grams [24] to filter out false positive candidate locations.
2. FEM verifies each candidate location with an efficient version of the banded Myers’ algorithm. We have implemented this bit-parallel algorithm with 128-bit registers and the SSE instruction set on a CPU to accelerate verification.
3. Finally, FEM generates alignment results in SAM format for valid mapping locations and puts them into a result queue.

## Results

### Experimental setup

We have implemented FEM in C++ and compiled it with GCC 4.8.5. All experiments have been performed on a Linux server with two Intel Xeon processors (E5-2650, 2.60 GHz), 64 GB of RAM, CentOS 7.2. We have thoroughly compared FEM with four state-of-the-art “all-mappers", which are designed to return all mapping positions of a read with respect to a given edit distance threshold: Hobbes3, BitMapper2, Bitmapper, and Masai. We have also included two popular best-mappers, GEM and BWA in the comparison. We exclude other all-mappers (such as mrFAST, mrsFAST [2], Razers3 and Yara [25]) in our comparison since it has been shown already previously in [26] that they do not perform as well as Hobbes3 and BitMapper in terms of either speed or accuracy.

In our experiments, we have used the human genome hg19 as reference. We evaluate the performance on both simulated and real short read datasets. Simulated reads are generated from hg19 using Mason [27] configured with default Illumina profile settings. We generate simulated reads of length 100bps. In addition, we use two real read datasets from NCBI SRA (accession numbers SRR826460 and SRR826471) with read lengths between 150 and 250bps. All mappers have been configured to exhaustively search for possible mapping locations with up to 4% of the read length as error threshold for simulated datasets and up to 3% for real datasets.

### Index construction and index size

We have tested the index construction time for hg19 for the hash-based mappers BitMapper, BitMapper2, Hobbes3, and FEM (using *l*_*step*_=2). Mappers based on BWT and the FM-index usually require significantly more construction time compared to hash-based mappers. Using a single thread, FEM requires 202.7s, BitMapper requires 627.6s, BitMapper2 requires 519.8s, and Hobbes3 requires 558.6s. Thus, FEM is fastest with a speedup of 3.1, 2.6, and 2.8 compared to Hobbes3, BitMapper, and BitMapper2, respectively.

Among these mappers, only FEM and Hobbes3 support parallel index construction. Using 32 threads, FEM requires 52.9s and Hobbes3 requires 249.9s to build the hg19 index. Index construction of FEM with multiple threads is thus an order-of magnitude-faster than BitMapper/BitMapper2 and 4.72 times faster than multi-threaded Hobbes3. The index construction time of FEM can be further reduced by increasing the value of *l*_*step*_; e.g. it takes 28.1s to build the the index for *l*_*step*_=3.

In terms of index size, Bitmapper uses 15 GB, BitMappers2 uses 4.9 GB, Hobbes3 uses 11 GB, and FEM uses 5.3 GB and 3.5 GB for *l*_*step*_=2 and 3, respectively. Thus, the index size of FEM is smaller than that of BitMapper2 when *l*_*step*_=3 and much smaller than that of BitMapper and Hobbes3. Users can configure *l*_*step*_ to a reasonable value when they have limited memory or use very large reference genomes. Table 1 summarizes the results for index construction.

**Table 1:** Index construction times (C-Time) and index sizes for hg19

Mappers	C-Time	C-Time	index size
	1 thread (s)	32 threads (s)	
FEM (*l*_*step*_=2)	202.7	52.9	5.3 GB
FEM (*l*_*step*_=3)	133.6	28.1	3.5 GB
Hobbes3	558.6	249.9	11 GB
Bitmapper	627.6	-	15 GB
Bitmapper2	519.8	-	4.9 GB

### Performance on simulated datasets

In order to evaluate the accuracy of the mappers, we used the Rabema benchmarking method [28], which is widely used in recent studies including [3, 4, 26]. Firstly, RazerS3 has been run in its full-sensitive mode to build the gold standard that contains all mapping locations with up to four errors. The gold standard is then used by Rabema to evaluate the accuracy of each mapper. The categories of sensitivity scores provided by Rabema benchmark include *all, all-best, any-best*. *All* represents all mapping locations within a given edit distance, *all-best* represents all mapping locations with the lowest edit distance, and *any-best* represents any mapping locations with the lowest edit distance.

Table 2 shows the number of mapped reads and accuracy of read mappers for 100,000 simulated reads with edit distance threshold 4. In the accuracy column, *total* denotes the accuracy of total mappings within the threshold and *ED i* denotes the accuracy of those mappings with edit distance *i*. FEM-vl and FEM-g denote FEM with variable-length seeding and group seeding, respectively. Both use *l*_*step*_=2.

**Table 2:** Rabema benchmarking results for mapping 100k simulated reads of length 100 bps to hg19

Mappers	Mapped	Accuracy $\left (\begin {array}{ll} & \text {ED 0 ED 1 ED 2} \\[-10pt] \text {total} & \\[-10pt] & \text {ED 3 ED 4}\\ \end {array}\right)$											
	reads	All[%]				All-best[%]				Any-best[%]			
FEM-vl	99997	100.00	100.0	100.0	100.0	100.00	100.0	100.0	100.0	100.00	100.0	100.0	100.0
			100.0	100.0			100.0	100.0			100.0	100.0	
FEM-g	99997	99.9705	100.0	100.0	100.0	100.00	100.0	100.0	100.0	100.00	100.0	100.0	100.0
			99.99	99.25			100.0	100.0			100.0	100.0	
Hobbes3	99997	100.00	100.0	100.0	100.0	100.00	100.0	100.0	100.0	100.00	100.0	100.0	100.0
			100.0	100.0			100.0	100.0			100.0	100.0	
BitMapper	99997	99.9999	100.0	100.0	100.0	100.00	100.0	100.0	100.0	100.00	100.0	100.0	100.0
			100.0	100.0			100.0	100.0			100.0	100.0	
BitMapper2	99997	99.9998	100.0	100.0	100.0	100.00	100.0	100.0	100.0	100.0	100.0	100.0	100.0
			100.0	100.0			100.0	100.0			100.0	100.0	
Masai	99995	99.9493	100.0	100.0	100.0	99.998	100.0	100.0	100.0	99.998	100.0	100.0	100.0
			99.97	98.73			100.0	97.33			100.0	97.33	
GEM	99994	98.6008	100.0	99.99	99.80	99.994	100.0	99.99	99.98	99.997	100.0	100.0	100.0
			89.97	70.24			100.0	96.00			100.0	96.00	
BWA	99990	92.2433	98.66	96.79	85.80	97.5195	97.46	97.70	97.35	99.985	100.0	99.97	99.93
			36.11	1.83			98.03	96.15			99.74	97.33	

Both FEM-vl and Hobbes3 achieve the highest accuracy score of 100.00%. BitMapper and BitMapper2 also return most of the mapping locations but are slightly worse than FEM-vl and Hobbes3. FEM-g only loses a few locations for the *all* category but maintains 100.00% accuracy scores for both *all-best* and *any-best*. Masai, GEM, and BWA cannot return mappings for all reads. Masai loses mappings in the *all-best* and *any-best* categories. GEM loses nearly 30% mapping locations for large edit distances. BWA performs worst and rarely returns mappings when edit distance is 4. Thus, we have decided to omit the inclusion of GEM and BWA for the performance evaluation on real datasets.

### Performance on real datasets

To test the mappers on real datasets, we extracted the first 5 million reads from SRR826460 and SRR826471 and mapped them against hg19. Table 3 and 4 show the results on 150 bps and 250 bps reads with the edit distance threshold set to 4 and 7, respectively. We have tested each mapper using 1, 8, 16, and 32 threads except for Masai, since it does not support multi-threading.

**Table 3:** Results for mapping 5 million real reads of length 150 bps to the hg19 (ED 4)

Mappers	1-thread time	8-thread time	16-thread time	32-thread time	Mapped reads
	(s)	(s)	(s)	(s)	(#)
FEM-vl	1370	195	101	78	4615727
FEM-g	1224	176	91	71	4615701
Hobbes3	2965	405	213	171	4615730
Bitmapper	1123	137	83	82	4615730
Bitmapper2	3405	467	250	232	4615829
Masai	6556	-	-	-	4615694

**Table 4:** Results for mapping 5 million real reads of length 250 bps to the hg19 (ED 7)

Mappers	1-thread time	8-thread time	16-thread time	32-thread time	Mapped reads
	(s)	(s)	(s)	(s)	(#)
FEM-vl	1085	150	78	56	4014477
FEM-g	893	126	64	48	4014476
Hobbes3	8442	1160	594	529	4014477
Bitmapper	1089	149	134	135	4014477
Bitmapper2	4957	680	353	310	4014474
Masai	11363	-	-	-	4014476

When mapping 5 million 150 bp reads against hg19 with the edit distance 4, BitMapper is slightly faster than FEM-g and FEM-vl when using less than or equal to 16 threads, but slower when using 32 threads. FEM-g is the fastest with 32 threads. BitMapper2 is around three times slower than BitMapper and returns incorrectly mapped reads across chromosome boundaries as mentioned in [26]. FEM-vl, FEM-g, BitMapper, and Hobbes3 return almost the same number of mapped reads. Masai loses 36 mapped reads, which is 0.00078% of mappable reads.

When mapping 5 million 250 bp reads against hg19 with edit distance 7, FEM-g is the fastest followed by FEM-vl. When using 32 threads, FEM-g and FEM-vl are 2.8 and 2.4 times faster than BitMapper and an order-of-magnitude faster than Hobbes3 and BitMapper2. Masai is the slowest. The numbers of mapped reads of different mappers are close together. FEM-g and Masai only lose one mappable reads and BitMapper2 loses 3.

In order to further compare scalability to bigger datasets, we have randomly extracted 20 million reads from SRR826460 and mapped them against hg19 using FEM, BitMapper, BitMapper2 and Hobbes3. Table 5 shows the results on mapping 150 bps reads with the edit distance threshold set to 4 and the number of threads set to 32. FEM-g and FEM-vl are still the fastest ones among the state-of-the-art all-mappers.

**Table 5:** Results for mapping 20 million real reads of length 150 bps to the hg19 (ED 4)

Mappers	FEM-g	FEM-vl	BitMapper2	BitMapper	Hobbes3
32-thread time/s	467	520	770	722	1189

**Fig. 6:**
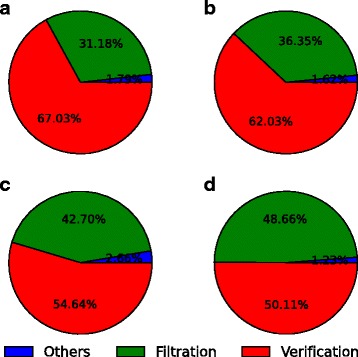
Percentage of runtime spent on different stages of FEM. **a**, **b**: When mapping the 150-bp real read dataset with FEM-g (**a**) and FEM-vl (**b**). **c**, **d**: When mapping the 250-bp real read dataset with FEM-g (**c**) and FEM-vl (**d**)

In addition, we have quantified the percentage of time spent on the different stages of FEM using the two datasets with read reads. The results shown in Fig. 6 show that the time on verification dominates the overall runtime. The filtration time of FEM-vl is slightly longer than it of FEM-g since seed extension is more expensive.

In terms of thread scalability, both FEM-vl and FEM-g are scalable for up to 32 threads on our machine. BitMapper2 and Hobbes3 only scale well for less than 16 threads. The runtime of BitMapper even increases when using 32 threads.

### Effects of the step size parameter

The step size to scan the reference when building the succinct hash index affects the performance of read mapping. By using a large step size, we can retain a small index size. Since both group seeding and variable-length seeding utilize the additional prefix algorithm [24] which selects *e*+2 seeds, then the upper bound of seed length *k*_*max*_ of a reads *R* is

6 $$ k_{max} =\left\lfloor \frac{\left| R\right| }{e+2} \right\rfloor.  $$

Thus, the upper bound of step size *l*_*max*_ is determined by the maximal seed length *k*_*max*_ and window size *k* for hash index as follows:

7 $$ l_{max} =k_{max}-k+1.  $$

**Fig. 7:**
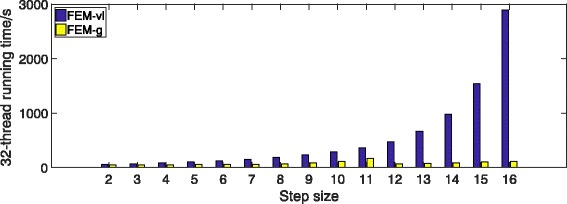
The mapping time of FEM-g and FEM-vl with different indexing step size *l*_*step*_

We run an experiment to evaluate the effect of the value of *l*_*step*_ on performance. In the experiment, we set the *k*-mer size (window size) as 12 and build the succinct hash index with different step sizes ranging from 2 to 16. We then measure the 32-thread run time for mapping 5 million 250 bp reads from real datasets that we have used to evaluate the speed. Figure 7 shows the results.

We can observe that the runtime of FEM with variable-length seeding becomes longer when increasing *l*_*step*_. This is because a larger step size reduces the search space when finding least frequent sub-seeds within each extended seed. Nevertheless, FEM-vl still outperforms BitMapper when the step size is less than or equal to 6, which means FEM-vl can reduce the index size by nearly 6 times without increasing the mapping time.

Furthermore, the runtime of FEM with group seeding is less affected by the step size. In order to analyze the reason, we comprehend the seeding algorithm as to select shorter *q*-mers from a read of length *r*^′^ called *sub-read*, where

8 $$ q=\left\lceil \frac{k}{l_{step}} \right\rceil  $$

and

9 $$ r'=\left\lfloor \frac{\left| r \right| -(l_{step}+k-1) }{l_{step}} \right\rfloor.  $$

The step size for *q*-mers in *r*^′^ is

10 $$ l_{step}^{\prime} = 1.  $$

We can observe that the running time drops when the step size equals to 6 or 12. When the step size is half or equals to the window size, according to Eq. 8, group seeding does not waste base-pairs to avoid overlap when selecting seeds in each seed group.

### Number of generated candidate locations

**Fig. 8:**
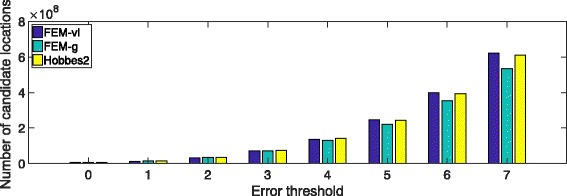
The number of candidate locations for FEM and Hobbes2 under different error thresholds

To analyze the filtration efficiency of our proposed seeding algorithm, we have counted the total candidate locations under different error thresholds varying from 0 to 7 when mapping 5 million 250-bp reads from the real dataset used in our speed evaluation. Since seeding algorithms adopt the seed selection algorithm in Hobbes2 [24], we have compared the total candidate locations generated by FEM-g, FEM-vl, and Hobbes2 (see Fig. 8).

We can observe that FEM-vl generates the least number of candidates when the error threshold is less or equal than 3. This is because the variable-length seeding algorithm extends seeds to choose sub-seeds with less total frequency than the frequency of fixed-length seeds chosen by FEM-g and Hobbes2. When the error threshold is greater than 3, the total number of candidate locations generated by FEM-g is the smallest. Since group seeding allows seeds in different seed groups to be overlapped, it is less effected by more limited seed pools. Thus, FEM-g can generate fewer candidates by choosing less frequent overlapped seeds in different seed groups.

## Discussion

FEM is an all-mapper that can return all mappings of a read with respect to a given edit distance. FEM achieves high speed at a low memory footprint by introducing a number of technical novelties:

1. A succinct hash index that significantly reduces the memory footprint compared to a classical *q*-gram index. The design and implementation of an efficient parallel algorithm allows us to build this index for a human reference genome within one minute.
2. The efficient seed selection algorithm called group-seeding is tailored towards the succinct hash index. It guarantees to return all mapping locations when only considering mismatches and maintains high accuracy if there are insertions or deletions.
3. To further tolerate indels, we propose another novel seed selection algorithm, variable-length seeding. Based on a seed length extension strategy, it achieves a high filtration efficiency and guarantees comprehensive accuracy in any cases.

Based on the specific seed selection algorithm, we have distinguished FEM as FEM-vl and as FEM-g. From our comprehensive evaluation of FEM compared to several state-of-the-art read mappers using both simulated and real genomic data, we can observe that FEM-vl returns all mapping locations and FEM-g can miss a few locations when considering insertions and deletions. As for the speed, FEM achieves a 2.8-fold speedup against BitMapper and an order-of-magnitude speedup against BitMapper2 and Hobbes3 while occupying significantly less memory. Furthermore, FEM is highly scalable and achieves a speedup of over 20 when using 32 threads on two Intel Xeon E5-2620 processors. Due to its low memory footprint and inherent parallelism, the FEM approach is a good candidate for implementation on modern accelerators such as CUDA-enabled GPUs and many-core architectures.

## Conclusion

Propelled by the continuing development of NGS technologies, the handling of large sequence datasets becomes increasingly challenging. Short read mapping is a performance-critical and compute-intensive step for a variety of NGS pipelines. In this paper, we have shown how a succinct hash index can be used as an efficient data structure for read mapping. Based on this data structure we have presented FEM, a fast and efficient read mapper designed to return all mapping positions of a read in a reference genome sequence with respect to an error threshold. Tailored towards the succinct hash index, FEM integrates two novel seed selection algorithms for generating a set of candidate locations. The group seeding algorithm can retrieve all mapping locations under hamming distances while the variable-length seeding algorithm additionally supports insertion and deletion. Our experimental results show that FEM substantially improves the performance of all-mappers in terms of both speed and accuracy. Furthermore, FEM scales when using multi-threading on common multi-core CPUs.

## Abbreviations

All: All mapping locations within a given edit distance
All-best: All mapping locations with the lowest edit distance
Any-best: Any mapping locations with the lowest edit distance
BWT: Burrows-Wheeler transform
bps: Base-pairs
CKS: Cheaper *k*-mer selection
C-Time: Construction time
DP: Dynamic programming
ED: Edit distance
FEM: The fast and efficient read mapper, which is the name of our read mapper
FEM-vl: FEM with variable-length seeding
FEM-g: FEM with group seeding
HBM: High banwidth memory
SRA: Short-read alignment
OPS: Optimal prefix selection
OSS: Optimal seed solver
